# Investigation of the Relationship Between Weaning Readiness and Maternal Depression: Cross-Sectional Online Survey

**DOI:** 10.3390/healthcare13050557

**Published:** 2025-03-05

**Authors:** Esra Sari, Irem Ozten Dalkiran, Nuray Arda, Haitham Jahrami

**Affiliations:** 1 Faculty of Health Science, Department of Midwifery, Van Yuzuncu Yil University, Van 65080, Türkiye; 2 Enstitute of Health Sciences, University of Health Sciences, Istanbul 34668, Türkiye; irem.ozten@outlook.com; 3 Enstitute of Health Sciences, Istanbul Medipol University, Istanbul 34815, Türkiye; nurayarda_dr@hotmail.com; 4 Department of Psychiatry, College of Medicine and Medical Sciences, Arabian Gulf University, Manama 329, Bahrain; haitham.jahrami@outlook.com; 5 Government Hospitals, Manama 329, Bahrain

**Keywords:** depression, maternal depression, midwifery, readiness for weaning, weaning

## Abstract

**Background/Objectives**: The initiation and continuation of breastfeeding are just as crucial as its conclusion. This study aimed to explore the connection between maternal depression and readiness for weaning. Although previous studies have examined maternal depression in the context of breastfeeding initiation and continuation, limited research has explored its role in weaning readiness. This study seeks to address this gap by investigating the psychological and emotional aspects of weaning in primiparous mothers. **Methods**: This descriptive cross-sectional study was conducted between May and July 2024 through online interviews. The descriptive information form, Readiness for Weaning Scale (RWS), and Beck Depression Inventory-II (BDI-II) were used for data collection. The sample included 83 primiparous mothers with a healthy pregnancy, no prior depression diagnosis or breastfeeding experience, and a baby aged 18 months or older. Statistical analyses were conducted to examine the correlation between RWS and BDI-II scores. **Results**: Participants’ mean age was 31.95 ± 5.25 years, and the average breastfeeding duration was 17.83 ± 9.79 months. Among the mothers, 63.9% reported readiness to wean, with a significant relationship between the RWS and readiness. Emotional challenges included sadness (62.7%), fear of damaging the maternal bond (45.8%), and feelings of deprivation (34.9%). The mean RWS score was 65.64 ± 11.31, while the mean BDI-II score was 9.67 ± 7.02. Higher depression scores were associated with lower readiness for weaning. **Conclusions**: Midwives play a crucial role in supporting mothers during weaning. This study highlights the need for targeted interventions to address maternal emotional well-being during this transition. Future research should explore culturally sensitive approaches to improve support mechanisms for mothers experiencing psychological distress while weaning.

## 1. Introduction

The emotional bond formed during pregnancy continues through breastfeeding, representing a unique mother–baby relationship [1,2]. The World Health Organization (WHO) recommends exclusive breastfeeding for six months and continued breastfeeding with supplementary foods until at least two years [3].

The breastfeeding process includes initiation, maintenance, and termination, with weaning representing the first psychological separation of mother and baby, which is often described as a challenging process [4].

Post-weaning depression is a mood disorder similar to postpartum depression and refers to the depressive symptoms that may emerge after a woman discontinues breastfeeding. Depression is characterized by persistent feelings of sadness and a loss of interest in activities that were previously enjoyable [5]. In a study conducted by Baser (2018) with mothers of infants aged two years and younger, a statistically significant relationship was identified between the duration and status of breastfeeding and postpartum depression, as well as breastfeeding history. Furthermore, the study found that mothers who breastfed for an extended period had significantly lower rates of postpartum depression, and the relationship between postpartum depression and breastfeeding for more than one year was also statistically significant [6].

Weaning is defined as the gradual cessation of breastfeeding and the transition to feeding with foods other than breast milk [7]. Reasons for discontinuing breastfeeding include the baby reaching two years of age, transitioning to supplementary foods, maternal fatigue, pregnancy, perceived milk insufficiency, medical issues, social pressure, or returning to work. Due to cultural differences, breastfeeding is discontinued in various ways. Common weaning methods involve altering the breast’s taste or appearance (e.g., applying pepper or tomato paste), using pacifiers, covering the breast, or using verbal strategies such as calling it “dirty” [1,8,9,10].

Breastfeeding impacts maternal mood, emotions, and stress [11]. Eye contact, touching, and caressing during breastfeeding strengthen the mother–baby bond, but weaning marks the end of this bond, making it a challenging yet necessary step for the growing baby [9,12]. According to the study conducted by Guleroglu, Uludag, and Cimke (2023) to determine the emotions and thoughts mothers experience during the weaning process, it was found that mothers experienced emotions and thoughts such as crying, sadness, guilt, depriving the baby of their right, breaking the bond with their baby, and anger during the weaning process [1].

The second phase of mammary gland regression is characterized by a decline in lactation-related hormone levels. Prolactin, the primary regulator of mammary gland function, fluctuates throughout lactation but eventually drops to levels typical of non-lactating states. Consequently, prolactin-driven production of parathyroid-related protein diminishes [13,14]. Additionally, after lactation ceases, the expression of the calcium-sensing receptor is also reduced [15]. However, despite the regression of mammary glands, serum serotonin levels, which are elevated during lactation, remain high even 21 days post-lactation [16]. These findings suggest that after lactation, other tissues may contribute to serotonin production.

Mothers often feel guilt, a sense of betrayal, or a loss of connection, which can trigger depressive symptoms. Hormonal changes during weaning, including reduced prolactin and oxytocin levels—key for milk production and maternal calm—are believed to contribute to mood swings [1,17,18]. Weaning without being ready leads to abrupt hormonal changes, increasing the risk of adverse effects, especially in mothers with a history of depression or those forced to wean prematurely [18]. Even mothers who wean gradually and feel ready may experience loss and sadness, as weaning ends the physical bond and a special chapter with their child [2,18].

A review of the literature reveals that there are very few studies explaining the relationship between weaning and maternal depression, highlighting a gap in this area.

There is a need for prospective, randomized controlled and cohort studies examining the relationship between weaning and maternal depression. This study aims to contribute to the literature and serve as a guide for future research in this field. The primary aim of this study, in line with these statements, was to investigate the relationship between weaning and maternal depression, and the secondary aim was to investigate the reasons for weaning and the methods used by mothers.

### Research Questions

**RQ_1_:** Is there a relationship between the cessation of breastfeeding and maternal depression?

**RQ_2_:** Does the cessation of breastfeeding increase depressive symptoms in women?

**RQ_3_:** What methods do women prefer when weaning?

**RQ_4_:** How do babies react when mothers stop breastfeeding?

**RQ_5_:** How do women feel during the weaning process?

## 2. Materials and Methods

### 2.1. Type, Place, and Time of the Study

The study, which was designed as descriptive cross-sectional research, was carried out between May and August 2024 via an online questionnaire (Google Forms) developed by the researchers. The online survey form was distributed to participants via social media platforms (Linkedln, Instagram, X, and WhatsApp).

### 2.2. Study Population and Sample

The population of the study consisted of mothers with babies over 18 months, and the sample consisted of women who had a healthy pregnancy, who had not been diagnosed with depression before pregnancy, who had a child aged 18 months and over, who had no previous breastfeeding experience, who were primiparous and between the ages of 18–49 years, and who agreed to participate in the study.

Attention has been given to the woman’s lack of previous breastfeeding experience and her first pregnancy, as it was believed that this would help better explain the relationship between readiness for weaning and maternal depression, without the influence of previous experiences. Additionally, it has been found that women with a history of depression are more likely to experience depression after weaning [19].

The sample of the study was selected using a criterion sampling method, consisting of 90 mothers. During the data review and cleaning process, data from seven women identified as outliers were excluded, and data analysis was conducted on 83 mothers.

After data collection and before data analysis, a post hoc sample size calculation was conducted. This revealed an effect size of 0.5, a Type I error rate of 0.05, and a power (1-ß) of 0.95. Thus, it was determined that the study’s sample size was sufficient.

### 2.3. Inclusion Criteria for the Study

The study included women who had a healthy pregnancy, had not been diagnosed with depression before pregnancy, had a child aged 18 months or older, had no previous breastfeeding experience, were primiparous, aged between 18 and 49 years, and provided their consent to take part in the study.

There is no ideal age for weaning; when given the opportunity, most infants will want to be breastfed until they no longer naturally require breast milk. This period generally spans from two and a half to seven years [17].

### 2.4. Exclusion Criteria for the Study

Women who decided to withdraw from the study after participating and who did not fill out or complete the data forms were excluded from participation in the study.

### 2.5. Data Collection for the Research

Research data were collected between May and July 2024 (2 months) using an online survey (Google Forms) created by the researchers. This form was shared with participants through stories, posts, and status updates on social media. The social media platforms used included Instagram, X, LinkedIn, and WhatsApp, where the access link to the online form was shared.

### 2.6. Data Collection Tools

Introductory Information Form (IIF): The researchers designed this form on the basis of a comprehensive literature review [1,9,10,20,21,22]. The Introductory Information Form included 29 questions addressing the sociodemographic (age, education level, employment status, perceived income status, etc.) and obstetric characteristics (the planning method of the most recent pregnancy, the mode of delivery, the gestational week at which the birth occurred, etc.) of the mother and infant/child. Additionally, questions related to maternal/newborn feeding and nutrition (such as whether information was received during the prenatal period regarding breastfeeding, what the mother feeds her baby, whether information was received about weaning, whether information was received from healthcare professionals regarding weaning, etc.) are included in this form.

Readiness for Weaning Scale (RWS): This scale was developed in 2023 by Turkmen et al. The scale consists of a total of 19 items and 4 subscales. These subscales are, in order: intention to wean, infant’s readiness, statements regarding weaning techniques, and mother’s readiness. Responses on the Likert-type scale range from 1: strongly disagree to 5: strongly agree. The scale can be applied to all mothers with infants aged 18 months and older. The total score that can be obtained from the scale varies between 19 and 95. The higher the scores obtained from the scale are, the higher the level of “Weaning Readiness”. The Cronbach’s alpha coefficient of the scale was determined to be 0.793 [23]. In this study, the Cronbach’s alpha coefficient of the scale was calculated to be 0.778.

Beck Depression Inventory-II (BDI-II): The scale was developed by Beck, Steer, and Brown in 1996. The validity and reliability of the scale were established by Dikmen in 2020. The scale comprises 21 items and follows a 4-point Likert format. The response options include statements such as “I have no more difficulty in making decisions than before”, “I cannot make decisions as easily as I used to”, “I find it very difficult to make decisions compared to before”, and “I cannot make up my mind about anything anymore”. The total score ranges from 0 to 63, with no reverse-scored items. A score ranging from 0 to 13 signifies minimal depression, 14 to 19 corresponds to mild depression, 20 to 28 indicates moderate depression, and 29 to 63 reflects severe depression. The Cronbach’s alpha coefficient for the inventory was calculated as 0.783 [24]. In this study, the Cronbach’s alpha coefficient of the scale was calculated to be 0.878.

### 2.7. Evaluation of the Data

The data were analyzed via the SPSS software package (version 25). The descriptive results of the study are presented together with the corresponding percentages in the case of nominal or ordinal variables. Continuous variables are presented with mean and standard deviation values. The Pearson Chi-Square test and Fisher’s Exact Test were used for categorical variables and the Mann–Whitney U test was used for non-categorical variables. Spearman correlation was applied for correlation evaluation of non-parametric values. A *p* value below 0.05 was considered significant [25,26,27].

### 2.8. Ethics Statement

Prior to the study, ethical approval number 92, dated 22 March 2024, was obtained from the Non-Interventional Clinical Research Ethics Committee of Tekirdag Dr. Ismail Fehmi Cumalioglu City Hospital. In line with the aim and scope of the study, information was provided to the mothers, and their consent was obtained. Necessary permission was obtained from the responsible authors for the use of the scales. At every stage of the study, adherence to publication principles and the principles of the Helsinki Declaration was maintained.

## 3. Results

This study included 90 mothers with babies aged 18 months or older and no prior depression diagnosis. After seven outliers were excluded, analyses were conducted on 83 mothers.

The mean age of the mothers was 31.9 ± 5.2 years (minimum–maximum: 20–44). When the type of pregnancy planning of primiparous mothers was examined, 84.3% of them had a planned pregnancy, and the mean gestational week at delivery was 38.7 ± 1.2 (minimum–maximum: 42–37). A total of 68.7% of the mothers had a cesarean section. The mean breastfeeding duration of the mothers was 17.8 ± 9.7 months (minimum–maximum: 1–41).

When Table 1 is examined, the highest educational level of the mothers is undergraduate (48.2%), and the lowest is primary school graduate (2.4%). The majority of mothers lived in urban centers (53%) and had a nuclear family structure (92.8%). A total of 54.2% of the mothers were employed. When the education levels of the spouses of the mothers were examined, the majority of them were undergraduates (51.8%) or employed (95.2%). The majority of the infants were exclusively breastfed for the first six months (72.3%). More women did not receive prenatal breastfeeding education (42.2%), and half of them did not receive information about weaning (50.6%). When the status of receiving information about weaning from health personnel was analyzed, 55.4% did not receive information from health personnel (Table 1).

Table 2 shows the methods used by mothers during the weaning process. The method most frequently used by mothers was a gradual reduction in breastfeeding (26.4%). A total of 25.2% of mothers reported that they ended breastfeeding via other methods, including direct cessation, not using any method, and the baby weaning themselves. Among these mothers, 18.1% stopped breastfeeding while providing supplementary food with a bottle, 7.2% preferred to talk, 4.8% opted to apply bitter and sour tastes such as garlic/tar/pepper/lemon, and 4.8% chose to apply black paint/nail polish/soot/patience stone (sabir stone).

When Table 3 was analyzed, 41% of the mothers stated that they wanted to wean because their baby/child reached the age of two, whereas 19.3% stated that they wanted to wean because the frequency of day/night breastfeeding was too high and because they felt tired.

As shown in Table 4, the behaviors of the infants during the weaning period were examined, and 54.2% of the infants understood, were calm, and weaned themselves. The percentage of mothers who experienced breast refusal was 20%. When mothers stopped breastfeeding, 34.9% felt as if they were betraying their baby, while 45.8% felt that their bond with their baby would be broken (Table 4).

When the effects of weaning on mothers’ feelings and thoughts were examined, it was found that the majority of mothers felt ready (63.9%), but 62.7% of mothers who weaned felt sad. In addition, half of the mothers stated that when weaned, they felt as if they had not done anything for their baby (49.4%) and that they felt that their bond would be broken (45.8%). A total of 34.9% of the mothers stated that they felt as if they were betraying their baby when they were weaned, and 34.9% stated that they felt as if they had taken away their baby’s rights (Table 5).

**Figure 1:**
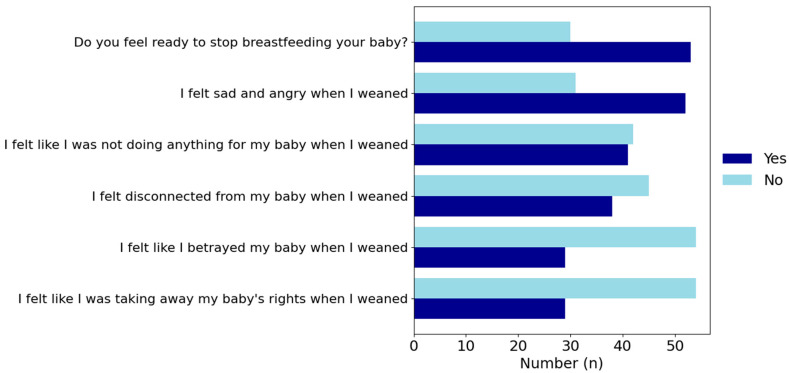
Weaning and emotional responses.

According to Figure 1, the weaning process generally involves mixed emotions for mothers, but it is observed that negative feelings are mostly minimal (Figure 1).

Table 6 shows a negative, low-level, and statistically significant relationship between mothers’ depression levels and their responses to questions about the weaning process (*p* < 0.05). The RWS had a total score of 65.6 ± 11.31 (range: 37–89), whereas the BDI-II had a total score of 9.67 ± 7.02 (range:1–32). It was determined that the relationships between mothers’ depression levels and the emotional states they experienced during the breastfeeding cessation process were low in magnitude, negative in direction, and statistically significant (Table 6).

## 4. Discussion

The study revealed that 72.3% of mothers exclusively breastfed their babies during the first six months. The World Health Organization (WHO) advises exclusive breastfeeding for the first six months, followed by the introduction of adequate complementary foods alongside breastfeeding until at least two years of age or beyond [28]. Globally, WHO data indicate that approximately 44% of infants are exclusively breastfed during the first six months [29]. According to the 2018 Turkish Demographic and Health Survey (TDHS) report, only 41% of infants under six months of age are exclusively breastfed [30]. When the results of the studies were compared, the rate of exclusive breastfeeding was found to be higher in this study. In addition, the average duration of breastfeeding of mothers was 17.8 months. According to the TDHS 2018 report, the median breastfeeding duration was 16.7 months, the average breastfeeding duration of mothers was 15.3 months in the study conducted by Oflu (2020), the average breastfeeding duration was 17.3 months in the study conducted by Altunel and Ozaydin (2022), and the research results are similar to those in the literature [9,30,31]. Furthermore, the rates found in the studies are all below the thresholds set by significant committees and boards, highlighting the importance of initiating, maintaining, and properly weaning breastfeeding.

In the present study, more than half of the mothers (55.4%) stated that they did not receive information from health personnel. In a study conducted by Caliskan and Balci (2022) with mothers with two-year-old children in Istanbul, 79% of the mothers did not receive any education on weaning [21]. In the study, conducted by Alsac and Polat (2018), the majority of mothers (89.1%) stated that they did not obtain information from healthcare professionals regarding weaning [22]. These studies support this study. According to the research findings, participants stated that they did not receive information from healthcare professionals regarding weaning, highlighting a gap in health centers. Possible reasons for this deficiency include heavy workloads, long working hours, and the perception that training on this topic is a waste of time.

Generally, gradual weaning by reducing breastfeeding step by step and abrupt weaning methods are used [1]. In this study, 26.4% of the mothers used the method of gradually decreasing breastfeeding, 33.6% used other methods (not using any method, baby weaning themselves, direct termination), and 18.1% provided supplementary feeding with a bottle. Weaning varies according to time and culture [30]. When the literature was examined, mothers used methods such as applying tomato paste/ketchup/hot pepper, using verbal expressions (ouchie, boo boo, dirty), sticking hair/wool, sticking tape/gum, applying vinegar/lemon, using a bottle, using a pacifier, and separating from the mother after weaning [10,21,31,32,33]. There are differences in the frequency of the methods used between this study and other studies. The fact that the majority of the mothers had an undergraduate degree and that the study was conducted with an online questionnaire may explain this difference. In a study conducted by Senol et al. (2023) with 195 mothers who were doctors, the most frequently used method for weaning was gradual weaning by talking to the child [8]. The results of the study conducted by Senol et al. support this study.

As a result of the study, 63.9% of the mothers were ready for weaning. Moreover, there was a negative and low-level significant relationship between the total RWS score and feeling ready for weaning (*p* = 0.31). Weaning is the first separation of mothers and babies and is a difficult process for both mothers and babies. Studies have shown that mothers experience emotions and thoughts such as crying, sadness, guilt, anger, taking away the baby’s rights, and breaking the bond with their baby during the process of termination of breastfeeding [1,20]. In this study, 62.7% of the mothers stated that they felt sad and/or angry when they stopped breastfeeding, 49.4% felt as if they were not doing anything for their baby/child, and almost half of them (45.8%) felt that the bond between them and their baby would be broken. The results of both studies support each other. A qualitative study conducted in Jordan revealed that mothers experienced emotions such as sadness, discomfort, worry, and anxiety arising from guilt. One mother expressed her feelings and thoughts as follows [12]:

- “When I weaned my three-month-old daughter, I was really upset and anxious because I had to go back to work. I felt like I was depriving her of something very important… She had the right to breastfeed… I am very sorry for this.”

Forty-one percent of the mothers wanted to wean because the baby reached/exceeded 2 years of age, 19.3% of the mothers wanted to wean because of the high frequency of day/night breastfeeding and because the mother felt tired, and 14.5% of the mothers wanted to terminate breastfeeding because the baby did not want to breastfeed. The behaviors exhibited by these mothers during the weaning process by their babies were as follows: understanding/calm/staying calm/baby, letting them go on their own/baby not wanting to suck (54.2%), breast refusal (24.1%), getting used to gradually letting them go (9.6%), attachment and denial (8.4%), and crying/anxious/irritable/wanting to stay in the arms (3.6%). A qualitative study by Abu Shosha (2020) in Jordan identified reasons for mothers deciding to wean, including pregnancy rediagnosis and returning to work. The themes of keeping the baby away from the mother, giving a bad taste to the nipples (applying salt, pepper, coffee, aloe vera), and making it difficult to access the breast (putting hair/cotton on the breast, taping, covering with clothes) were identified as methods to wean [12]. Healthcare providers can more effectively support mothers experiencing depressive symptoms during the weaning process by offering comprehensive education and information, individual counseling, psychological support, management of hormonal changes, and stress management techniques.

In this study, the Cronbach’s alpha coefficient for the RWS was 0.778, with subdimensional alpha values of 0.926, 0.816, 0.740, and 0.870. Turkmen et al. (2023) reported a mean total RWS score of 64.01 ± 11.31 (minimum–maximum: 19–88), whereas the mean score in this study was calculated as 65.6 ± 11.31 (minimum–maximum: 37–89), showing similar results between the two studies [23]. Additionally, the Cronbach’s alpha coefficient for the RWS in Turkmen et al.’s study was 0.793, indicating high reliability, with subdimension alpha values of 4.390, 3.343, 2.318, and 1.262. The findings of both studies are consistent and support one another.

Weaning is characterized by intense feelings of sadness and exhaustion. It is believed that the sudden changes in prolactin and oxytocin levels during weaning also affect emotions [19]. It was determined that the relationships between mothers’ depression levels and the emotional states they experienced during the breastfeeding cessation process were low in magnitude, negative in direction, and statistically significant. It was determined that the relationship between mothers’ depression levels and their readiness to cease breastfeeding was low in magnitude, positive in direction, and not statistically significant (*p* > 0.05). In a study conducted by Ystrom (2012), it was determined that prepartum anxiety and depression levels were associated with breastfeeding cessation, and breastfeeding cessation predicted an increase in postpartum anxiety and depression. Furthermore, it was found that prepartum anxiety and depression influenced the relationship between breastfeeding cessation and postpartum anxiety and depression [34].

## 5. Conclusions

Although postpartum depression after weaning is a relatively new research area, healthcare professionals and research teams believe that the emotional changes experienced during the weaning process, combined with hormonal fluctuations, can lead to psychological distress [19].

Weaning is as important as initiation and maintenance. Healthcare providers should organize educational programs for mothers about the process of weaning, practices of weaning, timing of weaning, and short- and long-term effects of weaning. These programs should be evidence-based, culturally sensitive, and integrated into routine maternal healthcare services to ensure accessibility and effectiveness.

Education sessions on the breastfeeding process have been ongoing for many years; however, when the literature is examined, it has been found that mothers still lack knowledge about breastfeeding. Similarly, structured and systematic education on weaning is insufficient. Addressing this gap through targeted interventions and counseling can improve maternal preparedness and emotional well-being during this transition.

Mothers need information and support provided by health professionals during the weaning period. Healthcare professionals, especially midwives, should play a proactive role in guiding mothers through the emotional and physiological aspects of weaning. Implementing structured support programs that include psychological counseling and peer support groups can help alleviate maternal distress and increase confidence in weaning decisions. Mothers are predicted to experience depressive symptoms during the process of weaning, and the likelihood of depressive symptoms decreases as weaning readiness increases.

Further research on this subject is needed. Future studies should focus on evaluating the effectiveness of different weaning support strategies, the role of healthcare professionals in facilitating the process, and the long-term psychological impact of weaning on both mothers and infants. Additionally, qualitative research exploring mothers’ personal experiences could provide deeper insights into their emotional and psychological challenges during weaning. By addressing these areas, this study contributes to developing comprehensive, practice-oriented solutions that enhance maternal and infant health outcomes.

## 6. Recommendation

Depression during the weaning process is typically a short-term concern, with most women experiencing an improvement as hormone levels stabilize. Healthcare professionals should extend their counseling services beyond initiating and maintaining breastfeeding to include support during the weaning process. In addition, to identify mothers who may be at risk for postpartum depression following weaning, a thorough prenatal history should be obtained, and family members should be educated about the potential emotional changes involved.

Post-weaning depression generally lasts for up to two weeks; however, if symptoms persist beyond this period, seeking assistance from healthcare providers is advisable. Special attention should be given to women with a history of depression, as they may require closer monitoring for postpartum depression following weaning.

Healthcare professionals should support women in recognizing the emotional challenges they may face during this period and assist them in confronting these difficulties. It is also essential to foster the woman’s self-confidence, reinforce her sense of self-worth, and help her develop coping strategies to manage even the most difficult circumstances.

## 7. Limitations

There is a potential for selection bias due to the online format of the survey, which may limit the diversity of the sample, and the exclusion of mothers with prior breastfeeding experience, which may affect the generalizability of the findings.

## Figures and Tables

**Table 1:** Sociodemographic, obstetric, and breastfeeding characteristics of the participants (n = 83).

Variables		Number (n)	Percentage (%)
Education level	Primary school	2	2.4
	Secondary school	4	4.8
	High school	17	20.5
	Undergraduate	40	48.2
	Postgraduate	20	24.1
Where they have lived the longest	Province	53	63.9
	District	24	28.9
	Village	6	7.2
Family type	Nuclear family	77	92.8
	Extended family	6	7.2
Employment status	Yes	45	54.2
	No	38	45.8
Income perception	Income less than expenditure	16	19.3
	Income equal to expenditure	43	51.8
	Income more than expenditure	24	28.9
Spouse’s education level	Primary school	3	3.6
	Secondary school	3	3.6
	High school	25	30.2
	Undergraduate	43	51.8
	Postgraduate	9	10.8
Spouse’s employment status	Yes	79	95.2
	No	4	4.8
How the most recent pregnancy was planned	Planned	70	84.3
	Unplanned	13	15.7
Delivery method	Vaginal delivery	24	28.9
	Forceps or vacuum delivery	2	2.4
	Cesarean section	57	68.7
Did you exclusively breastfeed your baby for the first six months?	Yes	60	72.3
	No	23	27.7
Did you receive breastfeeding education in the prenatal period?	Yes	35	42.2
	No	48	57.8
Did you receive information about weaning?	Yes	41	49.4
	No	42	50.6
Did you receive information about weaning from health personnel?	Yes	37	44.6
	No	46	55.4

**Table 2:** Methods used by the participants for weaning (n = 83).

Methods	Number (n)	Percentage (%)
Applying black paint, nail polish, soot, or patient stone (sabir stone) to the breast	4	4.8
Covering the breast with tape	4	4.8
Applying garlic, tar, tomato paste, pepper, lemon to the breast	4	4.8
Supplementary food with a bottle	15	18.1
Other methods (not using any method, baby weaning themselves, direct termination)	26	31.3
Gradual reduction in breastfeeding	24	28.9
By talking	6	7.2

**Table 3:** Reasons for weaning of participants (n = 83).

Reasons	Number (n)	Percentage (%)
Lactation insufficiency	7	8.4
Baby not wishing to breastfeed	12	14.5
Baby/child reaching/exceeding two years of age	34	41.0
Baby/child consuming supplementary food	4	4.8
Because of work	3	3.6
High frequency of breastfeeding during the day/night and the mother feeling tired	16	19.3
Lactation insufficiency + baby not wishing to breastfeed	3	3.6
Because of work + high frequency of breastfeeding during the day/night and the mother feeling tired	1	1.2
Pregnancy diagnosis	1	1.2
Breast problems (mastitis, nipple damage)	1	1.2
Baby starting biting	1	1.2

**Table 4:** Infants’ reactions when the participants wanted to wean (n = 83).

Methods	Number (n)	Percentage (%)
They did not want to breastfeed, understandingly, calmly	45	54.2
Habituation with gradual termination	8	9.6
Crying, restlessness, irritable, wanting to be held	3	3.6
Breast refusal	20	24.1
Commitment and denial	7	8.4

**Table 5:** Participants’ feelings and thoughts about weaning (n = 83).

Variables		Number (n)	Percentage (%)	*p*- Value
Do you feel ready to stop breastfeeding your baby?	Yes	53	63.9	0.12 *
	No	30	36.1	
I felt sad and angry when I weaned	Yes	52	62.7	0.021 *
	No	31	37.3	
I felt like I was not doing anything for my baby when I weaned	Yes	41	49.4	0.912
	No	42	50.6	
I felt disconnected from my baby when I weaned	Yes	38	45.8	0.442
	No	45	54.2	
I felt like I betrayed my baby when I weaned	Yes	29	34.9	0.006 *
	No	54	65.1	
I felt like I was taking away my baby’s rights when I weaned	Yes	29	34.9	0.006 *
	No	54	65.1	

* *p* < 0.05.

**Table 6:** Investigation of the relationships between participants’ Beck Depression Inventory-II total scores and their feelings and thoughts about weaning (n = 83).

		Depression
Weaning Readiness	r	−0.04
	*p*	0.664
Readiness for Weaning	r	0.14
	*p*	0.223
Feeling sad and angry after weaning	r	−0.28
	*p*	0.012
Feeling as if you have done nothing to the baby after weaning	r	−0.31
	*p*	0.004
Feeling disconnected from the baby after weaning	r	−0.23
	*p*	0.034
Feeling like betraying the baby after weaning	r	−0.26
	*p*	0.017
Feeling like you have taken away your baby’s rights after weaning	r	−0.34
	*p*	0.002

r: Correlation Coefficient (r); *p*: *p*-value.

## Data Availability

The original contributions presented in the study are included in the article; further inquiries can be directed to the corresponding author.
